# Determination of the factors affecting sexual violence against women in Turkey: a population-based analysis

**DOI:** 10.1186/s12905-021-01333-1

**Published:** 2021-05-05

**Authors:** Ömer Alkan, Hasan Hüseyin Tekmanlı

**Affiliations:** Department of Econometrics, Faculty of Economics and Administrative Sciences, Ataturk University, Erzurum, Turkey

**Keywords:** Domestic violence, Intimate partner violence, Sexual violence, Violence against women, Turkey

## Abstract

**Background:**

Sexual violence is one of the most investigated types of violence by national and international decision makers. The purpose of this study was to detect the factors that affect sexual violence against women in Turkey.

**Methods:**

In this study, a cross-sectional data set was employed from the survey titled the National Research on Domestic Violence against Women in Turkey, which was conducted by the Hacettepe University Institute of Population Studies. Binary logistic and probit regression analyses were used to determine the factors influential in women’s exposure to sexual violence.

**Results:**

The findings obtained from the analyses indicated that women’s exposure to sexual violence was influenced by a variety of factors including region, age, level of education, employment status, health condition, marital status, number of children as well as exposure to physical, economic, and verbal abuse. In addition, it was determined that the level of education, employment status, drug use, infidelity and other variables related to the husband/partner of the women who participated in the survey affected the women’s exposure to sexual violence.

**Conclusion:**

There remains a higher probability of exposure to sexual violence among women residing in rural and less developed regions. A decrease in the women’s level of education increased their probability of exposure to sexual violence. An increase in the women's age and an increase in the level of education of the women’s husbands/partners lowered the probability of their exposure to sexual violence. There was a higher probability of exposure to sexual violence among women who had experienced physical, economic, and verbal abuse.

## Background

Notwithstanding that it is a concept that changes according to time and socio-cultural structure, violence has become one of the most prominent issues in recent years [43]. Acts of violence are behavioral patterns that are internalized in the socialization process by new generations and passed down to other generations in this manner [5, 9]. One of the most common forms of violence against women is domestic violence committed by a husband or partner. Intimate partner violence (IPV), which is often referred to as domestic violence, takes various forms [55]. The intimate partner in such cases of violence is the husband/partner with whom the woman has sexual intercourse with or the father of the child she carried in her womb [38]. In the literature, non-physical violence is categorized into four different types emotional violence, psychological violence, social violence and economic violence [6, 53]. Physical and sexual violence refers to the form of violence regarding physical intervention against women [21, 38].

Violence is defined as the exertion of physical force upon another person from which there is a strong possibility that murder, injury, psychological damage or other negative changes will result. Furthermore, it is the use of physical force against oneself, another person or a group that deliberately causes or is likely to cause injury, death, psychological harm, negative development and/or deprivation. Violence can be physical, sexual or psychological in nature and can also include acts of deprivation or negligence [37]. There is no doubt that sexual violence is one of the most dangerous types of violence. Women all around the world face the risk of physical or sexual abuse by an intimate partner or another offender [35]. Sexual violence and IPV can lead to physical injuries, deterioration in mental health, and specific chronic physical ailments. In some cases such types of violence can even result in disability or death for some victims [18].

Sexual violence is a type of domestic violence that mostly occurs in marriage or intimate partnership scenarios [29]. Sexual violence and IPV are usually addressed together. In such cases, the concept of sexual violence by the intimate partner emerges and is very difficult to understand and measure as it includes both sexual violence and IPV [54]. While the most common manifestation of this form of violence is rape or sexual assault, women can also experience complicated situations such as coercion for sexual abuse or pornography, threats and blackmail [13].

Sexual violence takes place in all societies around the world, albeit under different definitions [33]. Sexual violence is defined as physically forcing another person to have sexual intercourse without their consent, having sexual intercourse because of the fear of the partner, and/ or being forced to perform a sexual act deemed humiliating [56].

Although sexual violence concerns both genders, women are more likely to be victims and in most cases the perpetrators are male and known by the victim. Moreover, children are particularly vulnerable to sexual abuse and girls are especially at greater danger at school [20]. Victims of sexual violence experience physical, social, mental, emotional, and sexual problems. Because of its severe psychological and sociological impact on the victim, sexual violence further escalates feelings of helplessness and weakness that can drain the victim's self-esteem and fuel a sense of vulnerability in the face of subsequent sexual violence [33]. From a more general classification, sexual violence results in the deterioration of mental and reproductive health and in the emergence of behavioral, social, and fatal consequences for the victim [17]. In addition, the expected results include hostility and blame on the basis of fear and anxiety in the short term and sleep disorders, depression, anxiety, obsession, acute stress disorder, mental retardation, and various significant health problems in the long term [46]. Sexual violence and forced sexual intercourse cause a range of gynecological and reproductive health problems such as HIV and other sexually transmitted infections, unwanted pregnancy, vaginal bleeding or infection, myoma, decreased sexual desire, genital irritation, pain during sexual intercourse, chronic pelvic pain and urinary tract infections [44]. Furthermore, they can causes short-term problems such as shock, fear, anxiety, panic, phobias, guilt, sleep disorders, eating disorders, and long-term psychological problems such as anxiety disorder, panic disorder, depression and suicide attempts [32]. Moreover, in extramarital affairs, some of the other potential outcomes are the failure of victims to form adult relationships, devalued expectations from marriage, and rejection by family and friends. In certain cultures there is even a possibility of beating or murdering the victim to protect the family honor [17].

On an international level, 30% of women experience some form of physical or sexual violence by their intimate partner during their lifetime. In cases of femicide, 38% to 50% of these acts are committed by the intimate partner. A vast majority of victims (55% to 95%) choose not to report the violence or take action to protect their rights [57], which is why it is difficult to obtain statistical records of sexual violence. The risk of facing sexual violence among women between the ages of 16 and 19 is four times higher than for other age groups and three times higher among women between the ages of 18 and 24. South Africa ranks at the top of the global list of countries with the highest rates of sexual violence or rape (132.4 incidents per 100 people) and is followed by, in descending the order, Botswana, Lesotho, Eswatini (Swaziland), Bermuda, Sweden, Surinam, Costa Rica, Nicaragua, and Grenada. With 1.5 incident per one hundred thousand people, Turkey is among the lowest ranking countries on this list [48].

In Turkey, the laws regarding women's rights and violence against women are noticeably modernized. However, most women have no idea of their rights, which may be due to the cultural norm that men own women, manhood is all about violence, and violence is just ordinary behavior. In addition, as Turkish people commonly adhere to Islamic norms, which order women to be submissive to men, it is generally accepted that rebelling against one’s husband is a sin [36]. As per the traditions and religious norms in Turkey, it is believed that violence, sexuality, and similar domestic matters should not be intervened with. As women widely hold the belief that, in marriage, the sexual needs of men must be met, they do not consider acts of sexual violence as criminal acts and never speak openly about these acts anywhere, let alone to judicial authorities [29].

The Regulation on the Implementation of the Law on the Protection of the Family enacted in 2008 regulated the measures to be taken against family members committing violence and the procedures and principles regarding the implementation of these measures. On combating violence against women, The Council of Europe Convention on Preventing and Combating Violence against Women and Domestic Violence, also known as the Istanbul Convention, was opened for signature in 2011 and came into force on 8 March 2012. This convention is one step ahead than former conventions in terms of the extended definition of violence against women and protecting and supporting women subjected to violence without considering stalking and cohabitation. With the Istanbul Convention, steps were taken to regulate the provisions of Law No. 4320 to prevent violence only in marriage. In order to eliminate the flaws in the implementation of this law, on March 8, 2012 Law No. 6284 on the Protection of the Family and the Prevention of Violence against Women entered into force. Law No. 6284 aims to protect women, children and victims of unilateral stalking who are married, engaged, divorced, in a relationship or who ended a relationship and are exposed to violence or in danger of being exposed to violence. By means of this particular law, the scope of the protective measure decisions aimed at preventing violence against individuals exposed to violence were extended and measures such as changing the identity information of the individuals concerned were included. Moreover, in order to be able to take preventive cautionary decisions against individuals who commit violence, the powers of the law enforcement officers in addition to judges were restructured in cases where delays were inconvenient [23]. The aim of these regulations is to prevent violence against women.

In a study conducted in Ankara, Turkey with 1178 women via a questionnaire, 31.3% of the respondents claimed to have experienced sexual violence from their husbands at least once, while 25.8% of the respondents claimed to have been physically forced to engage in sexual intercourse. Moreover, it was revealed that compared to the previous year, the ratio of exposure to sexual violence had increased by 15.9% [2]. In a study conducted in Turkey with 12,795 women via a questionnaire, 15% of married women between the ages of 15 and 49 reported to have experienced sexual or physical violence by their husband or intimate partner within the last 12 months. In another study carried out in Edirne, Turkey, it was determined that the rate of exposure to sexual violence was 6.3%. According to the findings of the study, the decrease in social support and the breakdown of marital relationship increased sexual violence [42]. In a study conducted on domestic violence including physical, verbal, economic, psychological and sexual violence in Eskişehir, Turkey, it was determined that the prevalence of sexual violence was 6.9%. [26]. In a study carried out in Isparta, Turkey, verbal/psychological, physical, emotional, economic and sexual violence were addressed within the scope of domestic violence and the rate of the exposure to sexual violence by husbands was determined as 38% [30]. In a study conducted in Konya, Turkey, it was found that approximately 38% of women were exposed to sexual violence [3].

Violence against women has long been a research topic in the scientific world and has been investigated in various aspects. In Turkey, the statistical methods or cross-sectional data sets for sexual violence are relatively insufficient compared to other types of violence. The present study aimed to fill this void in the literature by analyzing sexual violence via more specific variables in addition to cross-sectional data sets and demographic variables. In line with this purpose, the factors that influence sexual violence against women were modeled for Turkey via a rich data set.

## Methods

### Study design

The National Research on Domestic Violence against Women in Turkey is one of the most comprehensive studies conducted nationwide in order to understand the dimension, content, causes and effects and risk factors of domestic violence experienced by women in Turkey. It was conducted for the first time in 2008 in order to determine the different aspects and reasons of violence against women and to meet the requirement of collecting data on this issue. The National Research on Domestic Violence against Women in Turkey conducted in 2014 is significant in terms of reflecting the change regarding violence against women since the research conducted in 2008 [22, 23].

The research questionnaire was designed by taking into account the questionnaire applied by the WHO in the study titled “Multi-country Study on Women's Health and Domestic Violence against Women” [25]. New questions regarding country specific requirements, particularly targeting the monitoring of legal regulations, were also added to the questionnaire [22, 23].

### Setting

Within the scope of research on violence Turkey has been divided into 30 strata in order to ensure obtaining estimates at country level, urban/ rural level, at 12 and 5 regions level. Except for the Istanbul region, which is among one of the 12 regions, the distribution regarding urban and rural strata were at a rate of approximately 75% to 25% in the other regions. In Istanbul, approximately 5% of the households were selected from the rural areas. In the research, settlements with a population of 10.000 and above constituted the urban strata, and those with a population less than 10.000 constituted the rural strata. The sampling of the research was carried out using cluster sampling [22, 23].

The field study of the research conducted in 2008 started on 27 July 2008 and was completed on 29 September 2008 [22]. The field study of the research conducted in 2014 started on 8 April 2014 and was completed on 11 July 2014 [23].

### Participants

The National Research on Domestic Violence against Women in Turkey was conducted among women between the ages of 15 and 59. Women who were married, currently in a relationship, or had previously been in a relationship were included in the analysis within the scope of the present study. Single women who had never been in a relationship were excluded from the study. The demographic characteristics of the women who participated in the research are presented in Table 1.

**Table 1:** Demographic characteristics of the participants

Variables	Frequencies	%
Region		
TR1	1399	7.6
TR2/TR4	3075	16.6
TR3	1517	8.2
TR6	1614	8.7
TR5/TR7	2922	15.8
TR8/TR9	2962	16.0
TRC	1734	9.4
TRA/TRB	3288	17.8
Place of residence		
Rural	5158	27.9
Urban	13,353	72.1
Age		
15–24 years	2795	15.1
25–34 years	5855	31.6
35–44 years	4890	26.4
45–54 years	3630	19.6
55 + years	1341	7.2
Level of education		
Illiterate	3010	16.3
Elementary school graduate	8986	48.6
Secondary school graduate	1820	9.8
High school graduate	2975	16.1
University graduate	1717	9.3
Employment		
Unemployed	14,635	79.1
Employed	3876	20.9
Marital status		
Single	1433	7.7
Married	15,925	86.0
Widowed /divorced/separated	1153	6.2
Number of children		
No children	2689	14.5
One child	2901	15.7
Two or more children	12,921	69.8

### Variables

According to The National Research on Domestic Violence against Women in Turkey the following questions related to sexual violence were directed to the participating women: “Has your husband or one of your intimate partners exerted physical force to have intercourse with you?”, “Have you involuntarily engaged in sexual intercourse because of fear of potential threats from your husband or one of your intimate partners?” and “Has your husband or one of your intimate partners force you to perform a sexually demeaning or disgraceful act?” The women's experiences of sexual violence measured by these questions were used to create a dependent variable. If the participating women had experienced one or many of the above-mentioned cases, they were deemed to be a victim of husband/partner-inflicted sexual violence, however, if none of the cases were experienced, they were deemed to have not experienced sexual violence. Thus, the dependent variable code 1 was assigned to women who had experienced sexual violence and the dependent variable code 0 was assigned to those who had not.

### Data sources/measurement

In this study, the cross-sectional data of the National Research on Domestic Violence against Women in Turkey conducted in 2008 and 2014 by the Institute of Population Studies of Hacettepe University were used.

The survey questionnaires of The National Research on Domestic Violence against Women in Turkey were implemented by the research team. Ethical rules developed by the WHO were followed at every stage of the study, and various measures were taken to ensure the safety of both the participating women and the research team. Written consent was obtained from the participants before each interview. The researchers received traing on Ethical and Safety Rules, and conducted themselves in accordance with sensitivity of the subject at the beginning, during and after the interview process. The interviews were conducted with one woman from each household. In the event of there being more than one woman in the 15–59 age group in the household, the participating woman was chosen by using a random method in order not to ask the same questions to more than one woman in the same household. The researchers were very meticulous in ensuring that the interviews were conducted in an isolated environment. Moreover, all interviewers were trained on the confidentiality of the interviews. During the process of obtaining consent and providing information regarding the content of the study, the participants were informed that their answers would be kept confidential [22, 23].

### Bias

The data regarding women's history of exposure to sexual violence were the subjective responses of women. Thus, there was a decided risk that any data obtained by this method could be biased.

### Study size

In the research conducted in 2008, the questionnaire was completed by interviewing 12,795 women face to face and the rejection rate was 2.1%. The response rate for the interviews conducted with the women was 86.1% [22]. In the research conducted in 2014, the questionnaire was completed by interviewing 7462 women face to face, and the rejection rate was 4.4%. The response rate for the interviews conducted with the women is 83.3% [23]. The weights calculated in accordance with the sample design of the research were added to these data sets [22, 23].

### Quantitative variables

In this study, questions related to sociodemographic and economic characteristics and domestic violence were directed to the participants, and some of the variables thought to be influential were then integrated into the model. The variables of the socio-demographic and economic characteristics of the women were determined as survey year (2008, 2014), place of residence (rural, urban), age (15–24, 25–34, 35–44, 45–54, 55 and above), level of education (illiterate, elementary school graduate, secondary school graduate, high school graduate, university graduate), employment status (employed, unemployed), marital status (single, married, widowed/divorced/separated), health condition (bad/very bad, not bad, perfect/good), number of children (no children, one child, two or more children), and exposure to first-degree relative violence (no, yes). Factors related to the woman's husband/partner were husband/partner's level of education (illiterate, elementary school graduate, secondary school graduate, high school graduate, university graduate), husband/partner's job status (unemployed, public sector, private sector), husband/partner's alcohol usage (no, yes), husband/partner's gambling history (no, yes), husband/partner's drug usage (no, yes), husband/partner's infidelity (no, yes), exposure to husband/partner's economic violence (no, yes), exposure to husband/partner's verbal abuse (no, yes), and exposure to husband/partner's physical violence (no, yes).

The region variable was one of the independent variables in the study. At the basis of employing the Nomenclature of Units for Territorial Statistics (NUTS) in Turkey lies the obligation to establish Development Agencies [4]. Following the nomenclature of units for territorial statistics at Level 1, Turkey was divided into 12 regions. In order to obtain more meaningful results from the analysis, some of the regions were unified and then grouped into eight regions [16]. These regions and the cities within the regions are depicted in detail in Table 2.

**Table 2:** Statistical region units classification -Level 1

Code	Level 1	Provinces
TR1	İstanbul	İstanbul
TR2/TR4	West Marmara/East Marmara	Tekirdağ, Edirne, Kırklareli, Balıkesir, Çanakkale, Bursa, Eskişehir, Bilecik, Kocaeli, Sakarya, Düzce, Bolu, Yalova
TR3	Aegean	İzmir, Aydın, Denizli, Muğla, Manisa, Afyonkarahisar, Kütahya, Uşak
TR5/TR7	Western Anatolia/Central Anatolia	Ankara, Konya, Karaman, Kırıkkale, Aksaray, Niğde, Nevşehir, Kırşehir, Kayseri, Sivas, Yozgat
TR6	Mediterranean	Antalya, Isparta, Burdur, Adana, Mersin, Hatay, Kahramanmaraş, Osmaniye
TR8/TR9	West Blacksea/East Blacksea	Zonguldak, Karabük, Bartın, Kastamonu, Çankırı, Sinop, Samsun, Tokat, Çorum, Amasya, Trabzon, Ordu, Giresun, Rize, Artvin, Gümüşhane
TRA/TRB	Northeastern Anatolia/East Anatolia	Erzurum, Erzincan, Bayburt, Ağrı, Kars, Iğdır, Ardahan, Malatya, Elazığ, Bingöl, Tunceli, Van, Muş, Bitlis, Hakkâri
TRC	Southeastern Anatolia	Gaziantep, Adıyaman, Kilis, Şanlıurfa, Diyarbakır, Mardin, Batman, Şırnak, Siirt

All of the analyzed variables were categorical variables and  nominal or ordinary scales. Ordinal and nominal variables were described as dummy variables in order to observe the impact of the categories belonging to all the variables that would be integrated into the binary logistic regression and binary probit regression models [5, 8, 9].

### Statistical methods

Survey statistics in Stata 15 (Stata Corporation) were used to account for the complex sampling design and weights. Weighted analysis was performed. In addition, bivariate analysis was performed to identify the relationships between the dependent variable (exposure to sexual violence) and various factors. The bivariate relationships were predicted by evaluating significant differences using Pearson's chi-square tests for the categorical variables. Pearson's chi-square (χ2) not only provides information regarding the significance of the observed differences, but also provides detailed information about the categories of any differences found [7].

Binary logistic regression and binary probit regression analyses were conducted to determine the risk factors that were influential on the exposure to sexual violence. These particular analyses are used to study the relationship between the dependent variable and the independent variable(s) in cases where the result (dependent) variable has two options (binary/dichotomy). Binary logistic regression not only provides the opportunity to evaluate the statistical significance of each independent variable as a risk factor but also the opportunity to calculate the odds ratio. The cumulative logistic distribution function is used in the binary logit model and the cumulative normal distribution function (CDF) is used in the probit model. Although the logit and probit models have qualitatively similar results, the predicted values of the two models cannot be directly compared. The fact that normal CDF contains integral calculations is cited as a factor leading to a more widespread use of logistic CDF in practice [7].

## Results

### Characteristics of the participants

In this section, the frequency and percentages of the independent variables related to the model to be established are provided and interpreted. In Table 3, influential factors on women's exposure to sexual violence and the chi-square test statistics are provided.

**Table 3:** Distributions and chi-square test statistics of the factors influencing women's exposure to sexual violence

Variables	History of experiencing sexual violence		n (%)	χ^2^	*P*
	No	Yes			
Survey year					
2008	9849 (62)	1867 (71.4)	11,716 (63.3)	86.62	< .001
2014	6048 (38)	747 (28.6)	6795 (36.7)		
Health condition					
Perfect/good	7341 (46.2)	725 (27.7)	8066 (43.6)	503.99	< .001
Not bad	6533 (41.1)	1177 (45)	7710 (41.7)		
Bad/very bad	2018 (12.7)	711 (27.2)	2729 (14.7)		
First-degree relative violence					
No	14,258 (89.7)	2104 (80.6)	16,362 (88.4)	183.29	< .001
Yes	1637 (10.3)	508 (19.4)	2145 (11.6)		
Husband/partner's level of education					
Illiterate	538 (3.4)	191 (7.3)	729 (3.9)	343.68	< .001
Elementary school graduate	6434 (40.5)	1376 (52.7)	7810 (42.2)		
Secondary school graduate	2288 (14.4)	404 (15.5)	2692 (14.6)		
High school graduate	4044 (25.5)	438 (16.8)	4482 (24.2)		
University graduate	2578 (16.2)	204 (7.8)	2782 (15)		
Husband/partner's employment status					
Unemployed	2831 (17.8)	572 (21.9)	2403 (18.4)	43.37	< .001
Public sector	2404 (15.1)	294 (11.2)	2698 (14.6)		
Private sector	10,647 (67)	1748 (66.9)	12,395 (67)		
Husband/partner's alcohol usage					
No	12,770 (80.4)	1897 (72.6)	14,667 (79.3)	82.97	< .001
Yes	3120 (19.6)	717 (27.4)	3837 (20.7)		
Husband/partner's gambling history					
No	15,661 (98.6)	2456 (94)	18,117 (97.9)	232.762	< .001
Yes	226 (1.4)	157 (6)	383 (2.1)		
Husband/partner’s drug usage					
No	15,831 (99.7)	2576 (98.6)	18,407 (99.6)	65.180	< .001
Yes	45 (0.3)	37 (1.4)	82 (0.4)		
Husband/partner's infidelity					
No	14,819 (93.3)	2028 (77.6)	16,847 (91.1)	681.902	< .001
Yes	1059 (6.7)	584 (22.4)	1643 (8.9)		
Exposure to economic violence					
No	11,966 (76.6)	1211 (46.5)	13,177 (72.3)	1016.10	< .001
Yes	3646 (23.4)	1395 (53.5)	5041 (27.7)		
Exposure to verbal abuse					
No	10,141 (63.8)	365 (14)	10,506 (56.8)	2270.96	< .001
Yes	5956 (36.2)	2249 (86)	8005 (43.2)		
Exposure to physical violence					
No	11,276 (70.9)	451 (17.3)	11,727 (63.4)	2785.97	< .001
Yes	4621 (29.1)	2163 (82.7)	6784 (36.6)		

Based on the findings displayed in Table 3, the participants who took part in the research in 2008 constituted 63.3% of the sample. Women who experienced first-degree relative violence represented 11.6% of the sample. The chi-square test statistics of all the variables were determined to be significant.

Women whose husbands/partners were illiterate made up 3.9% of the sample, and whose husbands/partners were elementary school graduates made up 42.2% of the sample. Women whose husbands/partners were unemployed represented 18.4% of the sample, while whose husbands/partners worked in the private sector represented 67% of the sample. Women whose husbands/partners drank alcohol constituted 20.7% of the sample, and whose husbands/partners gambled constituted 2.1% of the sample. Women whose husbands/partners were unfaithful constituted 8.9% of the sample. Women who had experienced economic violence formed 27.7% of the sample, women who had experienced verbal abuse formed 43.2% of the sample and women who had experienced physical violence represented 36.6% of the sample.

### Multivariate analyses

In this study, the binary logistic regression and binary probit regression models were employed to determine the factors that influenced the likelihood of women experiencing sexual violence. The results of the estimated model are given in Table 4.

**Table 4:** Estimated model results of the influential factors in women's exposure to sexual violence

Variables	Binary logistic regression				Binary probit regression			
	β	Std. Error	95% CI		β	Std. Error	95% CI	
			Lower	Upper			Lower	Upper
Survey year (reference category: 2008)								
2014	− 0.303^a^	0.066	− 0.432	− 0.174	− 0.171^a^	0.036	− 0.242	− 0.100
Region (reference category: TR1)								
TR2/TR4	0.069	0.128	− 0.181	0.319	0.037	0.069	− 0.099	0.173
TR3	0.191	0.140	− 0.084	0.466	0.106	0.076	− 0.044	0.255
TR6	0.091	0.134	− 0.173	0.355	0.044	0.074	− 0.100	0.189
TR5/TR7	0.249^b^	0.123	0.008	0.490	0.145^b^	0.068	0.012	0.278
TR8/TR9	0.261^b^	0.127	0.011	0.510	0.144^b^	0.069	0.008	0.280
TRC	0.203	0.134	− 0.059	0.465	0.124	0.073	− 0.020	0.268
TRA/TRB	0.501^a^	0.124	0.258	0.744	0.283^a^	0.068	0.150	0.417
Place of residence (reference category: rural)								
Urban	− 0.112^c^	0.066	− 0.242	0.018	− 0.063^c^	0.036	− 0.134	0.009
Age (reference category: 15–24 years)								
25–34 years	− 0.236^c^	0.127	− 0.484	0.013	− 0.122^c^	0.069	− 0.257	0.012
35–44 years	− 0.305^b^	0.136	− 0.571	− 0.038	− 0.162^b^	0.074	− 0.308	− 0.016
45–54 years	− 0.085	0.143	− 0.365	0.195	− 0.039	0.077	− 0.190	0.113
55 + years	− 0.137	0.167	− 0.464	0.191	− 0.071	0.091	− 0.249	0.108
Level of education (reference category: university)								
Illiterate	0.374^c^	0.203	− 0.024	0.772	0.206^c^	0.107	− 0.004	0.416
Elementary school graduate	0.266	0.188	− 0.102	0.634	0.151	0.098	− 0.041	0.343
Secondary school graduate	0.206	0.204	− 0.194	0.606	0.124	0.108	− 0.087	0.335
High school graduate	0.150	0.190	− 0.222	0.523	0.081	0.099	− 0.112	0.274
Employment (reference category: unemployed)								
Employed	0.190^b^	0.077	0.039	0.341	0.103^b^	0.043	0.019	0.187
Marital status (reference category: widowed/divorced/separated)								
Single	− 1.650^a^	0.335	− 2.306	− 0.994	− 0.846^a^	0.162	− 1.164	− 0.528
Married	− 0.422^a^	0.105	− 0.628	− 0.216	− 0.246^a^	0.060	− 0.364	− 0.129
Health condition (reference category: bad/very bad)								
Perfect/good	− 0.293^a^	0.090	− 0.469	− 0.116	− 0.161^a^	0.050	− 0.259	− 0.063
Not bad	− 0.225^a^	0.081	− 0.383	− 0.066	− 0.123^a^	0.046	− 0.213	− 0.033
Number of children (reference category: no children)								
One child	− 0.279^c^	0.151	− 0.574	0.016	− 0.150^c^	0.080	− 0.307	0.006
Two or more children	0.016	0.136	− 0.251	0.283	0.007	0.073	− 0.136	0.149
Exposure to first-degree relative violence (reference category: no)								
Yes	0.307^a^	0.085	0.141	0.473	0.176^a^	0.048	0.081	0.270
Husband/partner's level of education (reference category: elementary school)								
Illiterate	0.438^a^	0.138	0.168	0.707	0.236^a^	0.077	0.085	0.386
Secondary school graduate	− 0.053	0.092	− 0.234	0.127	− 0.024	0.051	− 0.125	0.076
High school graduate	− 0.202^b^	0.092	− 0.383	− 0.022	− 0.115^b^	0.050	− 0.213	− 0.016
University graduate	− 0.127	0.140	− 0.401	0.147	− 0.078	0.075	− 0.225	0.068
Husband/partner's employment status (reference category: public sector)								
Unemployed	0.308^b^	0.119	0.075	0.541	0.176^a^	0.065	0.048	0.303
Private sector	0.106	0.103	− 0.097	0.309	0.058	0.056	− 0.051	0.167
Husband/partner's alcohol usage (reference category: no)								
Yes	0.069	0.077	− 0.082	0.220	0.036	0.043	− 0.048	0.119
Husband/partner's gambling history (reference category: no)								
Yes	0.216	0.156	− 0.089	0.521	0.146	0.091	− 0.033	0.325
Husband/partner’s drug usage (reference category: no)								
Yes	0.730^a^	0.281	0.179	1.281	0.435^a^	0.167	0.108	0.762
Husband/partner's infidelity (reference category: no)								
Yes	0.493^a^	0.085	0.327	0.660	0.299^a^	0.050	0.202	0.397
Exposure to economic violence (reference category: no)								
Yes	0.728^a^	0.065	0.601	0.855	0.402^a^	0.036	0.332	0.473
Exposure to verbal abuse (reference category: no)								
Yes	1.394^a^	0.086	1.226	1.563	0.710^a^	0.042	0.628	0.792
Exposure to physical violence (reference category: no)								
Yes	1.450^a^	0.082	1.289	1.611	0.766^a^	0.041	0.686	0.846
Constant	− 3.550^a^	0.316	− 4.169	− 2.932	− 1.914^a^	0.167	− 2.242	− 1.586

^a^*p* < 0.01, ^b^*p* < 0.05, ^c^*p* < 0.10; *Std. Error*: Standard Error; *CI*: Confidence Interval

### Average direct elasticities

The marginal impacts of the factors influencing women's history of sexual violence can be seen in Table 5. In the model created in the study, the existence of multicollinearity between the independent variables was also checked, and it was suggested that a variance inflation factor (VIF) value of five and above caused a moderate level of multicollinearity, while a level of 10 and above caused a high level of multicollinearity [5, 9]. The VIF results displayed in Table 5 indicated that there was none of the variables could cause a multicollinearity problem.

**Table 5:** Average elasticity values of the factors influential in women's exposure to sexual violence

Variables	Binary logistic regression		Binary probit regression		VIF	1/VIF
	Elasticity (%)	Std. Error	Elasticity (%)	Std. Error		
Survey year (reference category: 2008)						
2014	− 26.466^a^	0.058	− 34.445	0.074	1.040	0.958
Region (reference category: TR1)						
TR2/TR4	6.083	0.112	7.559	0.142	2.720	0.367
TR3	16.740	0.123	21.345	0.154	1.960	0.511
TR6	7.997	0.118	9.027	0.151	1.990	0.502
TR5/TR7	21.717^b^	0.108	29.054^b^	0.137	2.650	0.377
TR8/TR9	22.737^b^	0.111	28.876^b^	0.141	2.710	0.369
TRC	17.757	0.117	24.915	0.149	2.160	0.463
TRA/TRB	43.159^a^	0.108	55.149^a^	0.136	2.930	0.342
Place of residence (reference category: rural)						
Urban	− 9.691^c^	0.057	− 12.385	0.071^c^	1.140	0.874
Age (reference category: 15–24 years)						
25–34 years	− 20.356^c^	0.109	− 24.064^c^	0.134	3.100	0.323
35–44 years	− 26.390^b^	0.117	− 32.166^b^	0.146	3.320	0.301
45–54 years	− 7.295	0.122	− 7.462	0.149	3.140	0.318
55 + years	− 11.750	0.143	− 13.766	0.177	2.050	0.487
Level of education (reference category: university graduate)						
Illiterate	32.711^c^	0.179	41.427^c^	0.220	4.320	0.231
Elementary school graduate	23.362	0.167	30.789	0.203	5.730	0.175
Secondary school graduate	18.144	0.181	25.321	0.222	2.490	0.402
High school graduate	13.288	0.169	16.700	0.205	2.800	0.357
Employment (reference category: unemployed)						
Employed	16.433^b^	0.066	20.273^b^	0.083	1.150	0.872
Marital status (reference category: widowed/divorced/separated)						
Single	− 148.104^a^	0.314	− 178.415^a^	0.386	3.080	0.324
Married	− 35.862^a^	0.088	− 45.788^a^	0.106	2.150	0.466
Health condition (reference category: bad/very bad)						
Perfect/good	− 25.292^a^	0.078	− 31.430^a^	0.096	2.670	0.375
Not bad	− 19.346^a^	0.069	− 23.895^a^	0.088	2.370	0.422
Number of children (reference category: no children)						
One child	− 24.535^c^	0.132	− 30.591^c^	0.161	2.820	0.354
Two or more children	1.415	0.118	1.290	0.143	3.790	0.264
Exposure to first-degree relative violence (reference category: no)						
Yes	26.417^a^	0.072	33.965^a^	0.091	1.040	0.960
Husband/partner's level of education (reference category: elementary school graduate)						
Illiterate	37.007^a^	0.114	43.925^a^	0.137	1.160	0.863
Secondary school graduate	− 4.641	0.080	− 4.767	0.101	1.230	0.813
High school graduate	− 17.698^b^	0.081	− 23.014^b^	0.101	1.530	0.652
University graduate	− 11.069	0.122	− 15.610	0.151	2.110	0.473
Husband/partner's employment status (reference category: public sector)						
Unemployed	26.696^b^	0.104	34.702^a^	0.130	2.150	0.465
Private sector	9.286	0.091	11.719	0.114	2.200	0.455
Husband/partner's alcohol usage (reference category: no)						
Yes	5.999	0.067	7.050	0.084	1.180	0.850
Husband/partner's gambling history (reference category: no)						
Yes	18.574	0.132	28.190	0.171	1.070	0.934
Husband/partner’s drug usage (reference category: no)						
Yes	60.976^a^	0.225	78.987^a^	0.275	1.030	0.971
Husband/partner's infidelity (reference category: no)						
Yes	42.132^a^	0.071	56.495^a^	0.089	1.150	0.869
Exposure to economic violence (reference category: no)						
Yes	62.957^a^	0.056	76.978^a^	0.068	1.160	0.866
Exposure to verbal abuse (reference category: no)						
Yes	124.329^a^	0.079	137.174^a^	0.085	1.440	0.695
Exposure to physical violence (reference category: no)						
Yes	128.457^a^	0.074	144.131^a^	0.077	1.510	0.662
Pseudo R^2^	0.274	0.275				
Cox-Snell/M	0.201	0.202				
AIC	10,892.562	10,884.229				
BIC	11,197.012	11,188.679				
Log-likelihood	− 5407.281	− 5403.114				
Classification success	86.94	86.83				
*p* value	0.000	0.000				
N	18,150	18,150				

^a^*p* < 0.01, ^b^*p* < 0.05, ^c^*p* < 0.10; *Std. Error*: Standard Error; *VIF*: Variance Inflation Factor

Table 5 presents the goodness of fit of the estimated models, which revealed that the results obtained from both models were identical. The accurate classification of the binary logistics and binary probit models was computed as 86.94% and 86.83%, respectively. The fitness criteria for the model provided similar results for both models and were in an acceptable range for these kind of models.

According to the results of the binary logistics model presented in Table 5, women who participated in the research in 2014 were 26.47% less likely to face sexual violence from their husbands/partners compared to women who participated in the research in 2008. Women residing in the TRA/TRB region were 43.16% more likely to face sexual violence compared to women residing in the TR1 region. Women residing in the urban area were 9.7% less likely to face sexual violence compared to others. Women in the age group of 35–44 years were 26.4% less likely to face sexual violence compared to women in the age group of 15–24 years. Women who had never attended school were 32.7% more likely to face sexual violence compared to women who graduated from a university. Employed women were 16.4% more likely to face sexual violence compared to unemployed women. Women who had never been married were 148.1% less likely to face sexual violence from a husband/partner compared to women who were widowed/divorced/separated. Women with an excellent/good health condition were 25.3% less likely to face sexual violence compared to women with a poor/very poor health condition. Women who had children were 24.5% less likely to face sexual violence compared to women who had no children. Women who had been exposed to first degree relative violence, those whose husbands/partners used drugs, those whose husbands/partners had been unfaithful, those who had been subjected to economic violence by their husbands/partners, those who had been exposed to verbal violence by their husbands/partner, those who had been subjected to physical violence by their husbands/partners were 26.4%, 60.98%, 42.13%, 62.96%, 124.33% and 128.46% more likely to face sexual violence compared to other women, respectively. Women whose husbands/partners had not attended school were 37.01% more likely to face sexual violence compared to those whose husbands/partners were primary school graduates. Women whose husbands/partners were unemployed were 26.7% more likely to face sexual violence compared to those whose husbands/partner worked in the public sector.

According to the binary probit model results presented in Table 5, women who participated in the study in 2014 were 34.45% less likely to face sexual violence from their husbands/partners compared to the women who participated in the study in 2008. Women residing in the TRA/TRB region were 55.15% more likely to face sexual violence compared to women residing in the TR1 region. Women residing in the urban area were 12.39% less likely to face sexual violence compared to others. Women in the age group of 35–44 years were 32.17% less likely to face sexual violence compared to women in the age group of 15–24 years. Women who had never attended school were 41.43% more likely to face sexual violence compared to women who had graduated from university. Employed women were 20.27% more likely to face sexual violence compared to unemployed women. Women who had never been married were 178.42% less likely to face sexual violence from a husband/partner compared to women who were widowed/divorced/separated. Woman with an excellent/good health condition were 31.43% less likely to face sexual violence compared to women with a poor/very poor health condition. Women with children were 30.59% less likely to face sexual violence compared to women who did not. Women who had been exposed to first degree relative violence, those whose husbands/partners used drugs, those whose husbands/partners had been unfaithful, those who had been subjected to economic violence by their husbands/partners, those who had been exposed to verbal violence by their husbands/partners and those who had been subjected to physical violence by their husbands/partners were 33.97%, 78.99%, 56.495%, 76.98%, 137.17% and 144.13% more likely to face sexual violence compared to other women. Women whose husbands/partners had not attended school were 43.93% more likely to face sexual violence compared to those whose husbands/partners were primary school graduates. Women whose husbands/partners were unemployed were 34.7% more likely to face sexual violence compared to those whose husbands/partners worked in the public sector.

## Discussion

In this study, the factors that influenced sexual violence against women in Turkey were investigated by employing binary logistic and binary probit regression analyses. According to the results of the analyses, women who participated in the questionnaire in 2014 were less likely to experience sexual violence compared compared to women who had participated in the 2008 questionnaire. It can be stated that the legal regulations adopted for the prevention of violence against women 2008 had a substantial influence on this decrease [23].

Based on the model estimation results, it was determined that women living in the Western Anatolia/Central Anatolia regions, the Western Black Sea/Eastern Black Sea regions, and the Northeastern Anatolia/East Central Anatolia regions were more likely to experience sexual violence compared to women living in Istanbul. In parallel with these results, in a study conducted in Turkey, it was reported that women living in eastern and southeastern regions were more likely to experience sexual violence compared to those living in western regions [58]. It is generally accepted that the above-mentioned regions are at a lower development level than Istanbul. Moreover, urban development offers opportunities to stem the tide of violence against women in terms of forbearance, access to economic sources, assets, corporate assistance and support [39].

It was determined that women living in urban regions were less likely to experience sexual violence compared to those living in rural regions. The fact that women living in urban areas have easier access to the internet, newspapers, TV, and similar media outlets and possess a higher awareness of their legal rights and the fact the these conditions mostly apply to their husbands/partners could also be factors behind this conclusion. Similarly, in a study conducted with married women in Bangladesh it was determined that the women living in rural areas were exposed to sexual violence more frequently than those living in urban areas [40]. In a Togo-based study carried out among married women, it was determined that those living in cities were less likely to experience sexual violence [45]. The economic differences between the rural and urban areas may be influential in this result. Poverty, which is prevalent more commonly in rural areas than urban areas, gives rise to domestic stress and therefore, paves the road for violence. Furthermore, the factor of isolation in rural areas decreases the chances for women exposed to violence to access assistance and thus, increases the risk of violence towards women [10]. The general acceptance of the abuse of women in rural areas and the relevant social norms that prohibit abused women from speaking publicly and pursuing social support also reduce the likelihood of women reporting abuse to law enforcement authorities [49].

Based on the age variable, it was determined that women within the age ranges of 25–34 years and 35–44 years were less likely to experience sexual violence compared to those within the age range of 15–24 years. In a USA-based study, an increase in age was reported to move in parallel with a lowering risk of sexual violence [52]. Different from this situation, it was reported in a study conducted in East India that an increase in age further increased the risk of experiencing various types of violence [12]. The correlation between sexual violence and age is quite complicated. The changing economic contribution of women in society is a function of time and age. It has been stated that the influence of economic contribution and age along with the changing gender roles and types of violence are required to be researched longitudinally in detail [19].

In terms of the level of education variable, it was determined that the illiterate women were more likely to experience sexual violence compared to those who were university graduates. Similar to this finding, a study carried out in Nepal determined that uneducated women were at a greater risk of sexual violence from their intimate partners compared to educated women [11]. In a study conducted in Serbia, it was determined that women with a lower level of education were more likely to experience physical or sexual violence [24]. Findings from a study conducted in various regions of India indicated that a higher level of education decreased the possibility of women experiencing violence and sexual abuse from their intimate partners [47].

In addition, it was found that women who were employed were more likely to experience sexual violence compared to those who were unemployed. In parallel with this result, a study conducted in Indonesia reported that women with financial independence were more prone to experience sexual violence [28]. Moreover, it was concluded in a study conducted in India that married women were at high risk of being exposed to both physical and sexual violence [34]. Women who earn money can be perceived and considered as a threat to male dominance in patriarchal families according to the traditional power structure [34]. Arguably, in the event that women start earning money and contributing to the household income, they gain further independence and awareness of their rights and therefore, may challenge the traditional gender norms. Husbands who become anxious about safeguarding and maintaining their authority may respond to this situation with increased violence [19].

Women who were single or married were less likely to experience sexual violence compared to those who were widowed/divorced/separated. Similar to this finding, a study based in the USA, revealed that divorced and separated women had a higher likelihood of being exposed to sexual violence compared to married women [52]. In most cases it is unlikely that married men will practice sexual violence, as marriage is based on mutual consent [14].

Women with children were less likely to experience sexual violence compared to those with no children. In parallel with this result, a study conducted in Nepal found that the absence of children in a family increased the likelihood of a women’s exposure to sexual and intimate violence [11].

Women whose husbands/partners were illiterate faced a higher possibility of sexual violence than women those husbands/partners were elementary school graduates. Furthermore, women whose husbands/partners were high school graduates faced a lower possibility of sexual violence than those whose husband/partners were elementary school graduates. In a study carried out in Indonesia, it was concluded that women whose husbands had less than nine years of education faced a higher possibility of physical and sexual violence [28]. In addition, a study in Serbia revealed that the lower the husband/partner's level of education was, the higher the risk of physical or sexual violence risk for the woman became [24]. A study conducted in Ankara, Turkey concluded that, as partners’ level of education increased, an inverse fall occurred in the frequency of women’s exposure to sexual violence [2]. As partners with an education beyond secondary school education consider and perceive each other more as estimable, the probability of them exploiting and abusing each other could be to a lesser extent [1].

It is highly probable that women whose husbands/partners are unemployed are more likely experience sexual violence. It was reported in a study conducted in India that women whose partners were employed were less likely to be exposed to violence [34]. Similarly, it was concluded in a study conducted in Spain that the increase in male unemployment at a regional level increased the possibility of violence towards an intimate partner [50]. This is an anticipated consequence. Theoretically, male unemployment not only increases the stress but also results in further abuse and exploitation by undermining the control and economic security feelings of males and can create a further control impulsion on their partners [51]. At this point, unemployment insurance, welfare aid and entitlement programs designed to alleviate and diminish economic challenges and difficulties could be effective in reducing the violence towards intimate partners [27].

The present study determined that women whose husbands/partners used drugs were more likely to experience sexual violence. Similarly, in a study conducted in Serbia, it was determined that women whose husbands/partners took drugs were more likely to experience sexual or physical violence [24]. Worse still is the instances where the perpetrator drugs the drink of the victim to facilitate sexual assault. In such cases, although the effects may vary based on the type of substance, it prevents the victim from resisting against sexual assault and thus, facilitate sexual assault [15].

Women whose husbands/partners were unfaithful were more likely to experience sexual violence compared to those whose husbands/partners were faithful. In a study conducted in Indonesia, it was determined that women with unfaithful husbands/partners were exposed to higher rates of sexual violence [28]. Additionally, a Vietnam-based study revealed that women with unfaithful husbands/partners were more prone to experiencing several types of violence including sexual violence [31]. In a study conducted in Turkey, it was determined that women with unfaithful husbands/partners were exposed to nearly twice the rate of sexual violence those whose husbands/partners were faithful were exposed to [58].

In addition, women who experienced economic, verbal, physical abuse and violence from first-degree relatives were more prone to experience sexual violence compared to those who had never experienced such forms of violence. In a study conducted on various ethnic origins in Nigeria, it was determined that domestic violence was directly correlated with physical, psychological and sexual violence [41]. According to the results obtained from a study conducted in Ankara, Turkey, 57.6% of women who were exposed to sexual violence had also been subjected to physical violence, while 84.5% had also been subjected to economic violence, and 72.5% had also been subjected to emotional violence [2]. Furthermore, it was reported that experiencing physical violence in the past elevated the risk of exposure to sexual violence. Hence, it was concluded that violence against women is indeed a unity and violence in any form is part of a chain reaction that leads to violence in multiple forms.

This study had a number of limitations. Firstly, the data in the study were secondary data. The variables essential for performing statistical analyses consisted of the variables in the data set. However, some variables including profession and home ownership status, were missing in the data set and therefore could not be included in the analysis. Secondly, as the data was cross-sectional, the definite causal relationship between sexual violence and the related factors could not be inferred.

## Conclusion

This study was conducted with women living in Turkey and used data collected on two different dates. It is suggested that the obtained findings can lead the way for other studies that harness different econometric models and variables through employing cross-sectional data sets on sexual violence in Turkey. In relation to the envisaged measures to stop violence against women, these findings can also offer guidance to the related governmental bodies. By forming multivariate models that cover all of the aforementioned types of violence, analyses could be conducted in connection with the types of violence in the future.

## Data Availability

The data underlying this study is subject to third-party restrictions by the Turkey Statistical Institute. Data are available from the Turkish Statistical Institute (bilgi@tuik.gov.tr) for researchers who meet the criteria for access to confidential data. The authors of the study did not receive any special privileges in accessing the data.

## Abbreviations

VIF: Variance Inflation Factor
Std. Error: Standard error
